# A YOLO-Based Method for Head Detection in Complex Scenes

**DOI:** 10.3390/s24227367

**Published:** 2024-11-19

**Authors:** Ming Xie, Xiaobing Yang, Boxu Li, Yingjie Fan

**Affiliations:** College of Information Engineering, China Jiliang University, Hangzhou 310018, China; p22030854023@cjlu.edu.cn (M.X.); p23030854078@cjlu.edu.cn (B.L.); p23030854072@cjlu.edu.cn (Y.F.)

**Keywords:** head detection, complex scenes, context enhancement, non-maximum suppression, feature extraction

## Abstract

Detecting objects in intricate scenes has always presented a significant challenge in the field of machine vision. Complex scenes typically refer to situations in images or videos where there are numerous densely distributed and mutually occluded objects, making the object detection task even more difficult. This paper introduces a novel head detection algorithm, YOLO-Based Head Detection in Complex Scenes (YOLO-HDCS). Firstly, in complex scenes, head detection typically involves a large number of small objects that are randomly distributed. Traditional object detection algorithms struggle to address the challenge of small object detection. For this purpose, two new modules have been constructed: one is a feature fusion module based on context enhancement with scale adjustment, and the other is an attention-based convolutional module. These modules are characterized by high detection efficiency and high accuracy. They significantly improve the model’s multi-scale detection capabilities, thus enhancing the detection ability of the system. Secondly, it was found in practical operations that the original IoU function has a serious problem with overlapping detection in complex scenes. There is an IoU function that can ensure that the final selection boxes cover the object as accurately as possible without overlapping. This not only improves the detection performance but also greatly aids in enhancing the detection efficiency and accuracy. Our method achieves impressive results for head detection in complex scenarios, with average accuracy of 82.2%, and has the advantage of rapid loss convergence during training.

## 1. Introduction

With the rapid development of computer vision technology, significant progress has been made in research related to the field of object detection. Object detection aims to accurately identify and locate target objects of interest in images or videos. It has wide-ranging applications in fields such as autonomous driving, security surveillance, and building. In recent years, the rise of deep learning technology has brought revolutionary breakthroughs in object detection. Deep learning models such as convolutional neural networks (CNN) have shown strong capabilities in terms of feature extraction and representation learning, significantly improving the performance of object detection [1]. However, despite the numerous promising object detection algorithms that have been proposed, they still encounter significant challenges in practical applications. Firstly, the objects in the object detection task are of various types and shapes and may be affected by a variety of factors, such as light, occlusion, angle changes, and so forth. For example, on construction sites, they are affected by complex scenes, such as people’s heads being obscured by cranes and the issue of construction site lighting being dim and bright intermittently at night. Complex scenes are typically defined as images or videos containing a large number of densely packed and occluded objects. The dense and occluded distribution of objects makes object detection tasks in such scenes more complex and challenging. Therefore, the goal of this study is to propose a solution to improve object detection in complex scenes so as to improve the performance of algorithms in these scenarios. Of course, this requires a more efficient and accurate head detection algorithm. This necessitates improving the robustness and generalization of object detection models. We hope that this research will promote the further development of object detection technology and provide more reliable and efficient solutions for practical applications. In addition, it is expected that this research will serve as a valuable reference, providing ideas for subsequent research in the field of object detection.

**Figure 1:**
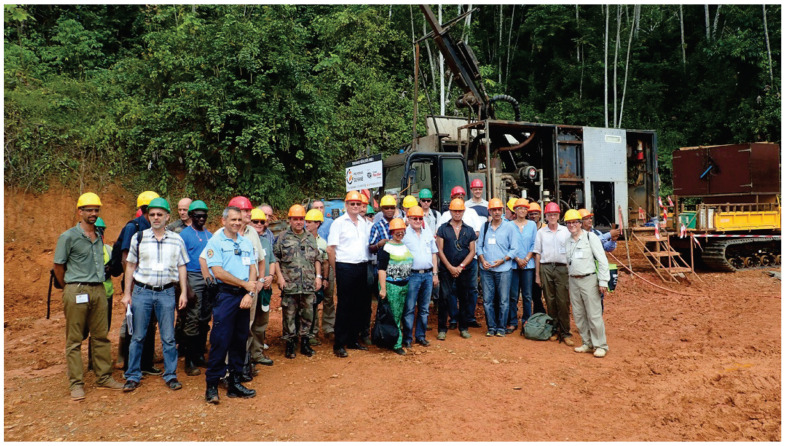
Complex scenes in construction sites.

The study of object detection in complex scenes is of considerable practical importance and value in terms of its potential applications. Firstly, in real life, complex scenes are ubiquitous, such as shopping malls with heavy foot traffic, stations, stadiums, and other public places, as well as densely populated urban roads with heavy vehicle traffic. In such scenes, the accurate detection of objects is of significant value for a number of applications, including security monitoring in buildings, crowd management, traffic diversion, and so forth. Secondly, the ongoing advancement of computer vision technology has driven the widespread adoption of object detection algorithms. These algorithms have become increasingly prevalent in a multitude of fields, including security monitoring, autonomous driving, and building, among others. However, in complex scenes, traditional object detection algorithms frequently encounter difficulties in attaining the desired performance, due to the presence of occlusion and the dense distribution of objects. It is for these reasons that this study was designed to facilitate the broader application and development of object detection technology by addressing the challenges of detecting objects in complex scenes. In addition, this study has significant theoretical implications. By delving into an in-depth exploration of the intricate object detection problem that arises in complex scenes, we can enrich and refine the theoretical system of object detection algorithms. In conclusion, this study aims to address the issue of object detection in complex scenes, which has significant practical, applied, and theoretical implications. Complex scenes contain a large number of objects, including both identifiable and unidentifiable ones, and these objects overlap with each other. This configuration often renders the object detection task more complex and challenging. As shown in Figure 1, such complex environments can further complicate detection. In recent years, with the continuous development of deep learning technology, researchers have conducted extensive research and exploration on the problem of object detection in complex scenes. The initial object detection algorithms primarily relied on manually designed features and classifiers for the identification and localization of objects. Examples of such algorithms include the use of Haar features [2] and the combination of HOG features with SVM classifiers [3,4,5]. However, these algorithms frequently encounter challenges when it comes to accurately detecting and locating objects within complex scenes. This is particularly challenging when there are significant occlusions and overlaps among the objects.

With the rise of deep learning technology, object detection algorithms based on convolutional neural networks have made significant progress in complex scenes. To illustrate, algorithms such as Faster R-CNN [6], YOLO [7,8], and SSD [9] have been shown to enhance the accuracy and efficiency of object detection through the construction of multilevel feature pyramids and region proposal networks. The Only Look Once (YOLO) series [10,11,12,13,14,15,16] is the main neural network used for real-time object detection tasks, widely used for feature detection [17,18]. Nevertheless, the challenges in object detection remain substantial in complex environments. A complex environment typically refers to scenes where factors such as variable lighting, cluttered backgrounds, diverse object shapes, and dynamic scene changes interact. These factors often lead to a decline in detection performance, especially noticeable when detecting small objects. A complex environment is a subset of complex scenes, specifically referring to scenes with a large number of overlapping or closely distributed objects. The detection of small objects in complex scenes is often challenging because they may be obscured by larger objects or background elements. Moreover, the close arrangement of objects makes it challenging for traditional object detection algorithms to effectively distinguish between closely spaced objects during feature extraction, directly affecting the detection accuracy. Conventional object detection algorithms typically exhibit robust performance when handling larger objects. However, their effectiveness diminishes considerably when confronted with smaller objects, primarily due to insufficient feature information, constraints on the spatial resolution, and interference from noise. This not only impacts the localization of objects but can also result in missed detection or false positives, thereby affecting the overall reliability of the system. Conducting thorough research into the attributes and determinants of small object detection within intricate and complex scenes is therefore of paramount importance. It is essential to explore effective strategies that can enhance the overall efficacy of object detection. Future research should concentrate on refining the feature extraction methodologies, bolstering the models’ robustness, and integrating data from multiple sensors to tackle the challenges associated with detecting small objects within intricate scenes. This will facilitate the wider adoption of object detection technology across practical applications.

To address the issue of small object detection in complex scenes and improve the model’s multi-scale fusion capabilities for better performance and accuracy, we fused convolutions of different sizes, enabling the system to detect small objects more effectively. Inspired by the Context Augmentation Module (CAM) [19] and the CSPStage [20] in Demo-YOLO, a Context Enhancement with Scale Adjustment Module (CESA) was designed. The reason for incorporating adaptive processing is that, although features can be obtained for small objects after convolution with different sizes, they cannot be used directly without further processing. This design ensures high computational efficiency and low latency, allowing the system to process both high-level semantic information and low-level spatial information with equal priority. This facilitates the fusion of multi-scale features and provides excellent fusion capabilities for the combination of low-level and high-level features.

Research findings indicate that the attention mechanism is highly effective in identifying the information that is most pertinent to the current task from a vast array of data. Furthermore, it has been shown to significantly enhance the performance of neural network architectures [21,22,23,24]. The attention mechanism in deep learning is an approach that emulates the human visual and cognitive system, enabling neural networks to concentrate on pertinent aspects of the input data as they are processed [25]. The attention mechanism is pivotal in the realm of small object detection. It hones in on crucial features, thereby heightening the visibility of diminutive objects, which in turn simplifies their identification. This mechanism effectively diminishes the clutter of the background and mitigates the interference from surrounding objects, thereby augmenting the precision of detection. Furthermore, it amalgamates features across various scales, enriching the depiction of small objects within feature maps at multiple levels. The attention mechanism also adjusts the weights based on the content of the input image, thus enhancing the model’s resilience. Additionally, it expedites the learning process, enabling the model to rapidly assimilate the characteristics of small objects and thereby bolstering the overall detection efficacy. The Simple Attention Module (SimAM) [26] and standard convolution are thus referenced to design a lightweight, parameter-free convolution module specifically designed for CNNs, with the objective of improving the model’s performance in image processing tasks without adding to the computational burden and model complexity. The conventional convolutional module, known as Conv-BN-SiLU (CBS), has been replaced by an innovative module that we have named the Simple Attention Conv-BN-SiLU (Sim-CBS). The Sim-CBS module consists of five components: standard convolution, feature extraction, local self-similarity computation, attention weight generation, and feature map weighting.

While reviewing the literature, it was discovered that appropriate adjustments to Non-Maximum Suppression (NMS) can improve the accuracy. In the field of object detection, NMS is a commonly used technique for the selection of the optimal object frame among those with greater overlap to reduce the issue of repeated detection. The fundamental aspect of NMS is the computation of the overlap metric between two frames, which is typically employed as an Intersection over Union (IoU) measure. In the NMS process, a set of overlapping object boxes is initially sorted according to confidence or other ratings. Subsequently, the IoU values of the remaining boxes in relation to the current box are evaluated individually, commencing with the highest-rated box. If the IoU value of a box exceeds the predefined threshold, it is discarded; otherwise, it is retained. This ensures that the final selected frame covers the object as accurately as possible without overlapping. Given the significant improvement of the SIoU in dealing with target occlusion in crowded environments, we decided to incorporate the SIoU into our algorithm [27]. The advantages of the Structured Intersection over Union (SIoU) [28] are numerous: (1) improved accuracy—by using the IoU to evaluate the overlap, the location of the object frame can be determined with greater precision; (2) simplicity and effectiveness—the implementation of NMS is relatively simple and effective, and it has been widely used in a variety of object detection algorithms; (3) adaptability—NMS is not dependent on the use of specific object detection algorithms and can be integrated with a wide range of models that have different structural configurations; (4) robustness—with suitable IoU threshold settings, it is possible to maintain consistent performance across different data sets and scenes and reduce the rate of false and leakage detection; (5) the principles and advantages of the SIoU as an overlap metric in NMS make it an indispensable and important technique in the field of object detection.

## 2. Improved Object Detection Network Model

### 2.1. YOLO Framework

YOLO represents an advanced object detection algorithm that not only inherits the efficiency and speed attributes of its predecessors but also further enhances the performance, especially within the realm of intricate and complex scenes [29]. The algorithm’s network and techniques improve the detection precision and maintain high processing speeds. This unique combination of features allows the algorithm to effortlessly meet the diverse and demanding requirements of real-world applications. The network structure is depicted in the accompanying diagram. Firstly, the algorithm employs a lightweight network structure, such as MobileNetV2, which enables the model to operate effectively on mobile and edge computing devices while maintaining high performance. The lightweight design not only reduces the model’s computational complexity but also decreases its storage requirements, thereby facilitating deployment and usage in practical applications. Secondly, a novel backbone network design is introduced, which serves to enhance the extraction of features and effectively improve the model’s detection performance. By increasing the resolution of the features and incorporating more contextual information, the model is able to gain a more nuanced understanding of the objects within the image. The aforementioned backbone network design demonstrates enhanced resilience in the processing of objects of disparate sizes and shapes. Furthermore, YOLO employs a cascading and pyramid approach, thereby empowering the algorithm to process objects of varying sizes. Regarding the network structure, the object detection task is divided into two distinct sub-tasks: classification and localization. The algorithm is able to better handle objects of varying sizes due to the fact that each sub-task has its own dedicated network path. This design boosts the efficiency of object detection in visually cluttered scenes. Regarding the loss function, YOLO has undergone optimization as well. The novel loss function considers object positional and class information and includes additional regularization terms to improve the model stability and generalization. This allows YOLO to converge better during training and exhibit higher performance on the test set.

The YOLO object detection network enhances the accuracy and speed using innovative techniques like lightweight structures, new backbones, cascading, pyramid methods, and optimized loss functions [30,31]. This makes YOLO widely applicable in various real-world application scenes, particularly in fields such as autonomous driving, security surveillance, and medical image analysis, where it holds significant potential [32,33,34].

**Figure 2:**
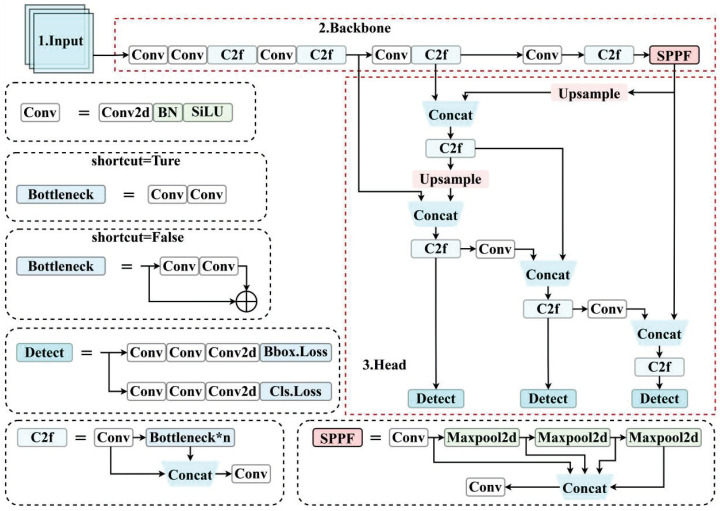
YOLOv8 detection structure. * represents multiplication.

This backbone network generally consists of a sequence of convolutional layers, arranged in a tiered structure. Each layer is responsible for extracting increasingly abstract features from the input image, as illustrated in Figure 2.

#### 2.1.1. Backbone Network in YOLO

The backbone network in YOLO serves as the foundation for feature extraction, playing a pivotal role in capturing hierarchical representations of input images. The backbone network generally consists of a sequence of convolutional layers, arranged in a hierarchical structure. Each layer is responsible for extracting increasingly abstract features from the input image.

Key Components. (1) The backbone network is composed of several layers of convolutional filters, which scan across the input image to extract pertinent features. These layers operate on the raw pixel values of the input image and progressively learn to capture more complex patterns and structures. (2) Downsampling layers such as max-pooling or strided convolutions are interspersed throughout the backbone network to reduce the spatial dimensions of the feature maps while increasing the receptive field. Downsampling helps in capturing features at different scales and resolutions, enabling the network to detect objects of varying sizes. (3) Some backbone architectures, such as ResNet, incorporate skip connections or residual connections between layers. These connections facilitate the flow of gradients during training and mitigate the vanishing gradient problem, allowing for deeper networks to be trained more effectively. (4) In certain backbone architectures, feature fusion techniques may be employed to combine features from multiple layers or branches of the network. Feature fusion enhances the network’s ability to capture both low-level details and high-level semantics, leading to richer feature representations.

Common Architectures. (1) Darknet is a lightweight neural network architecture specifically designed for YOLO models. It consists of a series of convolutional layers followed by downsampling operations, with skip connections between selected layers to aid in feature propagation. (2) ResNet is a popular backbone architecture, known for its effectiveness in training very deep networks. It introduces residual connections that enable the network to learn residual mappings, facilitating the training of deeper architectures without suffering from degradations in performance. (3) EfficientNet is a family of models that achieve state-of-the-art performance while maintaining computational efficiency. It leverages compound scaling to simultaneously scale the depth, width, and resolution of the network, resulting in models that are highly efficient and effective for various tasks, including object detection.

Our experimental goal was to improve and optimize the object detection algorithm, rather than merely focusing on using the latest version. As such, we chose YOLOv8 as the starting model, prioritizing its performance and potential for structural optimization over the novelty of the version. Therefore, YOLOv8 was selected as a practical and efficient choice, particularly since there is no pressing need for an upgrade at this stage.

In summary, the backbone network in YOLO plays a critical role in feature extraction, capturing hierarchical representations of the input images that are subsequently used for object detection. By leveraging advanced architectures and techniques, the backbone network enhances the model’s ability to detect objects accurately and efficiently.

#### 2.1.2. Head Network in YOLO

The detection head in YOLO is a crucial component, responsible for generating predictions based on the features extracted from the backbone network. The system utilizes feature maps derived from various tiers of the feature pyramid as input, executing object detection through the prediction of bounding boxes, class probabilities, and object scores.

Key Components. (1) The detection head typically consists of a series of convolutional layers that operate on the feature maps obtained from the backbone network. These layers apply learned filters to extract high-level features relevant to object detection tasks. (2) Pooling layers are often included in the detection head to reduce the spatial dimensions of the feature maps while preserving important features. This downsampling process helps in capturing semantic information and improving the computational efficiency. (3) At the end of the detection head, there are output prediction layers, responsible for predicting the bounding boxes, class probabilities, and object scores. These predictions are made for a predefined set of anchor boxes spanning various aspect ratios and scales. (4) The output prediction layers predict offsets for each anchor box to adjust its position and size relative to the corresponding grid cell. These offsets, along with the coordinates of the anchor box, are used to compute the final bounding box predictions. (5) In addition to bounding box regression, the detection head predicts the probability distribution over different object classes for each bounding box. This is usually accomplished using Softmax activation on the class scores. (6) YOLO predicts an object score for each anchor box, indicating the likelihood of the box containing an object of interest. This score helps in filtering out irrelevant detections and improving the precision of the model.

### 2.2. YOLO-HDCS Network

**Figure 3:**
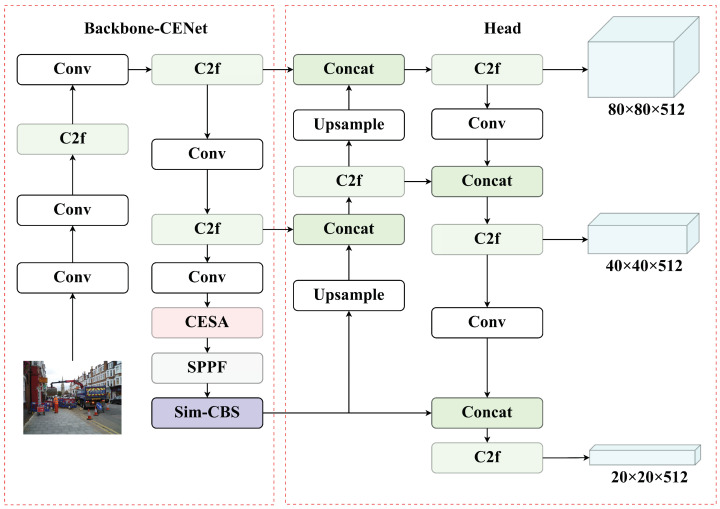
Overview of the network architecture for YOLO-HDCS.

This paper introduces a novel head detection algorithm, YOLO-HDCS, based on the YOLO framework. We propose a novel backbone network, designated as Backbone-CENet. The network is principally based on two key modules, the CESA and the Sim-CBS, both of which are proposed in this paper, as illustrated in Figure 3. When facing the challenges of complex and crowded scenes, the optimization of detection models becomes particularly important, as these scenes often require models to have higher accuracy and robustness. We have developed innovative strategies with unique benefits, significantly improving the detection model’s performance and effectiveness using various technical approaches.

The first improvement is the CESA module. In particular, we conducted in-depth research and practice on the challenging issue of small object detection in complex and crowded scenes. We innovatively fuse convolutional kernels of various sizes, a strategy aimed at fully utilizing the differences in the ability of convolutional kernels of different sizes to capture features. Small convolutional kernels excel in capturing fine details, which is crucial for the detection of small objects, while larger convolutional kernels provide a broader contextual view, improving the overall detection accuracy. Our design draws inspiration from CAM and CSPStage, but with a key difference: the features obtained from the convolution of different kernel sizes cannot be directly used without further processing. By effectively integrating the strengths of both small and large convolutional kernels, our system significantly enhances small object detection, even in dense or complex environments. To fully realize this capability, we incorporate an adaptive processing stage to refine and optimize the features for accurate identification and localization.

The second improvement is the Sim-CBS module. In this paper, we draw inspiration from SimAM and standard convolution to design a lightweight, parameter-free convolution module for CNNs. The objective is to enhance the performance of the model in image processing tasks without introducing an additional computational burden or model complexity. Specifically, we introduce Sim-CBS to replace the standard convolutional module, CBS. This new module, Sim-CBS, consists of five components: standard convolution, feature extraction, local self-similarity computation, attention weight generation, and feature map weighting.

The third improvement is NMS, which is a method of filtering out duplicate bounding boxes. To ensure the accuracy of the detection process, the output of the detection network typically includes multiple overlapping frames for the same object. NMS is applied to eliminate redundant boxes, keeping only the most relevant one. In traditional NMS, the box with the highest score is kept, while lower-scoring overlapping boxes are suppressed. However, the SIoU works differently. Instead of removing overlapping boxes, the SIoU adjusts the scores of the other boxes based on their overlap with the highest-scoring box. When two boxes have a high level of overlap, the SIoU lowers the score of the second box, making it "weaker", rather than removing it entirely. In summary, the SIoU improves upon the traditional NMS by modifying the scores of overlapping boxes, instead of directly suppressing them, which allows more relevant boxes to be retained.

#### 2.2.1. CESA

**Figure 4:**
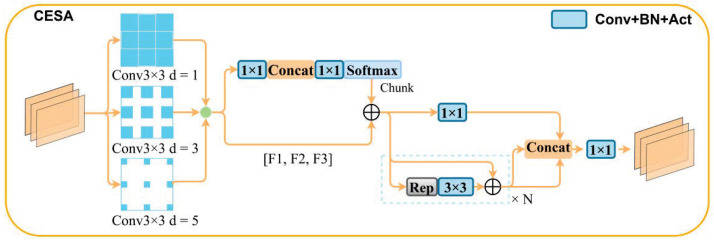
CESA feature network fusion module.

CESA is a feature fusion module for small object detection. For an illustration, please refer to Figure 4. The integration of multi-scale convolutional features, from the highest to the lowest levels, is embedded within the feature pyramid network to enrich the contextual information. The integration of channel and spatial feature refinement mechanisms aims to suppress the emergence of conflicts during multi-scale feature fusion, thus protecting small objects from being swamped by contradictory information. To further improve the training, a data augmentation method called copy–reduce–paste is introduced. This increases the contribution of tiny objects to the loss during the training process, thereby ensuring more balanced training.

The newly developed CESA feature network is designed with three key objectives:

1. First, it aims to significantly improve the efficiency with which features are utilized throughout the network;
2. Second, it enhances the network’s ability to capture fine details in the input data, enabling the more accurate detection and recognition of subtle patterns;
3. Third, it accelerates the model’s convergence during training, allowing for faster learning and the optimization of the network parameters.

To strengthen the multi-scale detection capabilities, the fusion of features across different scales is essential. In previous detection networks, features at different scales often showed significant depth discrepancies. For example, high-resolution features used to detect small objects tend to have relatively shallow depths, which can negatively impact the small object detection performance. Directly fusing features of varying depths typically leads to unsatisfactory results, so it is important to retain more original information during the initial processing. To address this, dilated convolutions with varying dilation rates are used to gather contextual information at different receptive field sizes. Specifically, 3 × 3 convolution kernels with dilation rates of 1, 3, and 5 are applied to the input, providing richer contextual information within the FPN.

The CESA module includes the following steps.

In CESA, a global feature pyramid is introduced with the objective of extracting richer semantic information through multi-scale feature fusion. The module is designed for computer vision tasks and aims to enhance the deep learning model’s ability to understand objects or scenes in an image by incorporating contextual information. The following section outlines the operational procedure of CESA. The input feature maps are received from the preceding layer of the convolutional or feature extraction network. The feature maps in question contain representations of features extracted from the original image at varying degrees of abstraction.

Context awareness: At the core of this lies the context-aware mechanism, which enhances the representation of the feature maps by incorporating additional contextual information. Such additional information may be global contextual features, local region-related information, or other forms of contextual information.

Feature Fusion: In the CAM module, the contextual information is integrated with the original feature map. This process typically entails the utilization of convolutional operations or attention mechanisms, with the objective of facilitating the efficient integration of information from disparate sources. The objective of feature fusion is to augment the expressive capacity of the feature map, thereby enabling the model to more effectively comprehend the semantic and structural nuances present in the image. The further processing of the fused feature maps is conducted with the objective of enhancing the representation of the object or scene of interest in the image. This may entail an increase in the resolution of the feature map, the enhancement of the semantic relevance of the features, or the strengthening of the responses of specific regions. Subsequently, the enhanced feature map is generated as the output.

The CESA module receives input features from the preceding layer, which is typically the output of the backbone network. The aforementioned features comprise a variety of semantic and spatial information pertaining to the image in question. Cross-stage connectivity is a technique that enables the transfer of information between layers in a neural network. In this phase, the CESA module divides the input features into two sections. One section passes through one or more convolutional layers and becomes the backbone path, while the other section bypasses the backbone path and serves as the skip path. Processing of the Backbone Path: The convolutional layers on the backbone path are employed for the purpose of extracting high-level semantic information from the input features. These convolutional layers typically possess a substantial receptive field, enabling the capture of global information and abstract features within the image. Branch Processing: Features on the branch paths are processed directly, bypassing a portion of the trunk path, and they retain low-level spatial information about the input features. This information is instrumental in retaining the details and positional information of the object. Feature Fusion: The features of the trunk paths and branch paths are, to some extent, complementary, because the former emphasize semantic information while the latter retain spatial information. CESA combines the two through a feature fusion operation to obtain a richer and more balanced representation of the features. Output Features: After feature fusion, the features generated by CESA are passed to the next network stage for further processing. These features have better semantic and spatial information, which is beneficial for subsequent object detection tasks.

The REP structure usually consists of the following steps. Feature Fusion: Firstly, the REP structure receives features from the backbone and branch paths in CESA. These features have already been combined by the cross-stage linking and feature fusion steps in CESA, and the task of the REP structure is to further enhance the feature representation. The REP structure uses residual linking to sum the features of trunk paths and tributaries. This residual joining helps to retain more feature information and prevent information loss and gradient vanishing. Next, the REP structure performs an enhancement operation on the summed features. This enhancement operation can take various forms, such as convolution and attention mechanisms, in order to further improve the discriminability and representational capabilities of the features. Ultimately, the REP structure typically executes pooling operations on the enhanced features to diminish their dimensions and computational demands, while simultaneously bolstering the stability and invariance of these features. Common pooling operations include average pooling, maximum pooling, and others.

CESA is introduced into YOLOv8 and compared with the C2f structure. We achieve higher accuracy, as expected. It can fully exchange high-level semantic information and low-level spatial information. It is a powerful object detection architecture that can effectively improve the accuracy and performance of object detection by combining global and local features.

The adaptive method is a type of adaptive fusion method. In the fields of computer vision and deep learning, it typically refers to adaptive feature fusion strategies. These methods aim to enhance the model performance by dynamically selecting and integrating feature information from different levels or scales. In this way, the methods adapt to different data and task requirements. This approach significantly improves the model performance, optimizes the efficiency of feature utilization and accelerates the speed of experimental iteration. The depth and complexity of a network have a direct impact on the model’s performance. Enhanced fitting capabilities often lead to the faster convergence of the loss function, as the network can extract information from more layers, thereby accelerating the optimization process. Specifically, assuming that the input size can be represented as (bs, C, H, W), we can obtain spatial adaptive weights of (bs, 3, H, W) through convolutions, concatenations, and Softmax operations. The three channels correspond one-to-one with the three inputs, and context information can be aggregated into the output by calculating the weighted sum. Using this method requires more features to be processed. The advantages and characteristics of the adaptive method are as follows: (1) by fusing multi-layer features, the model can obtain more comprehensive and rich feature information, thereby improving the accuracy of predictions; (2) different feature layers contain information at different scales and semantic levels, and adaptive feature fusion enables the model to have stronger robustness to objects of different scales and shapes; (3) it allows the model to adaptively select useful information from the feature layers based on the actual situation of the region of interest, enhancing the flexibility and generalization ability of the model.

Below are the three functions of the CESA module.

1. Introducing contextual information can enhance the model’s understanding of the relationships between the targets and backgrounds in images, thereby improving the accuracy of object detection. Context enhancement techniques improve the target localization and classification accuracy by capturing both global and local information. The aim of this technology is to improve the model’s performance in processing complex scenes, allowing it to analyze targets or events in images more accurately. By incorporating global information, the model can better understand the semantics and context of the image, reducing false positives and false negatives. Analyzing the enhanced contextual information, the model can precisely locate the target position, as the contextual information provides the spatial relationship between the target and surrounding objects. By introducing diverse contextual information during training, the model can learn a wider range of visual features and semantic concepts to deal with various scenes. The generated heatmaps show the distribution of the model’s attention, enhancing the model’s visualization effect and credibility. Context enhancement takes into account multiple factors for a more comprehensive understanding of the image content and more accurate predictions.
2. The adaptive receptive field allows neural networks to automatically adjust the size of the receptive field based on the scale and position of the target, enhancing the ability to process targets of different scales and improving the generalization and adaptability. The receptive field is the range of responses of neurons to the input image or feature map. When the target is large, the receptive field expands to capture the overall features; when the target is small, the receptive field contracts to preserve the details. Through dynamic adjustment, the module takes into account the position of the target and the background, improving the detection accuracy. During training, the module learns to adapt to inputs of different scales and complexities, reducing the dependence on manual parameters and enhancing its universality and flexibility. Compared to fixed receptive fields, adaptive receptive fields adjust their size as needed, effectively utilizing the computational resources and reducing the computational complexity and storage requirements. This feature is compatible with modern deep learning frameworks, supporting end-to-end training and deployment and simplifying the model design and optimization process.
3. End-to-end training can simplify the model construction process and optimize the capture and utilization of contextual information. When combined with modern object detection frameworks, end-to-end training optimizes the performance while ensuring efficient and stable deployment. End-to-end training is a comprehensive training method from input to output, integrating all components of the system into a single model and using backpropagation algorithms for global optimization. This method eliminates the interface challenges of traditional multi-stage processing, such as feature extraction and candidate box generation in object detection, directly optimizing the overall performance through a single model. It avoids information loss in staged optimization, enhances the synergy of components through global loss function optimization, reduces manual intervention, and enhances the model’s automation and versatility. Through global information transfer and feedback, it reduces overfitting and improves the generalization ability. In addition, end-to-end training simplifies model design and increases the directness and efficiency of system implementation.

#### 2.2.2. Sim-CBS

**Figure 5:**
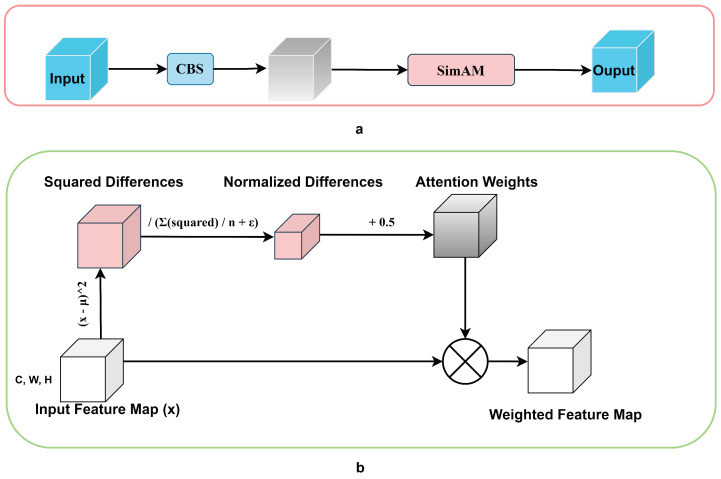
Simple attention Conv-BN-SiLU module. (**a**) A standard convolutional module with an added attention mechanism. (**b**) The SimAM network structure.

Despite various efforts to optimize CNN networks, such as reducing the convolutional kernel sizes, the 1 × 1 regular convolutional module still requires a significant amount of computational effort as the network becomes more complex. As is well known, the attention mechanism can focus on the most interesting detection areas, thereby performing dense detection in these areas and thus improving the object detection capabilities of the network. However, in complex scenes, we not only need to focus on the detection areas but also to minimize the computational complexity of the network as much as possible. Therefore, we need to incorporate a lightweight and simple attention mechanism. Regular 1 × 1 convolutional modules still demand substantial computation as the network deepens, which can be problematic. In this paper, we use a lightweight, parameter-free attention mechanism specifically designed for CNNs. Our aim is to enhance the performance of the model in image processing tasks without adding an additional computational burden or model complexity. As shown in Figure 5, the proposed module replaces the standard convolutional block (CBS). It comprises five components: standard convolution, feature extraction, local self-similarity computation, attention weight generation, and feature map weighting.

SimAM aims to fully explore the self-similarity information inside the feature map and then dynamically adjust the weights of different regions in the feature map, thereby enhancing the model’s ability to capture key information in the image. This mechanism not only enhances the model’s performance in visual tasks but also significantly optimizes the model’s computational efficiency and resource consumption, providing a new perspective for the application of deep learning in the field of image processing. Traditional attention modules require the introduction of learnable parameters to model feature dependencies, which increases the complexity and may lead to overfitting. SimAM, on the other hand, generates attention weights using feature map information, without the need for additional parameters, simplifying the model structure and reducing the computational burden. The attention weights dynamically change, adaptively adjusting to different image content, which improves the model’s generalization ability. SimAM enhances the feature expression of highly self-similar regions, suppresses redundant noise, focuses on key information, and improves the accuracy of the model in tasks such as image classification, object detection, and segmentation. SimAM, as an attention mechanism based on self-similarity perception, represents a significant advancement in the application of deep learning in the field of image processing. In addition to streamlining the model structure and reducing the computational cost, the dynamic weight adjustment mechanism enables SimAM to focus on key information, thereby enhancing the overall performance.

The operational methodology of Sim-CBS can be delineated as follows. First, the image features are processed by standard convolution, and then the CNN extracts the feature maps of the input image. These feature maps contain high-level semantic information of the image. For each pixel in the feature map, SimAM calculates its similarity with the neighboring pixels. This step is based on the principle of the local self-similarity of images, which posits that neighboring pixels usually have strong similarity. Based on the calculation of the local self-similarity, SimAM generates an attention weight for each pixel. This weight reflects the importance of the pixel in the feature map. Finally, the generated attention weights are multiplied with the original feature maps to obtain weighted feature maps. These weighted feature maps highlight the key areas in the image more prominently, which helps to improve the performance of subsequent tasks.

The model is parameter-free. The introduction of additional parameters is not necessary for Sim-CBS, thus avoiding any increase in the complexity and computational cost of the model. This algorithm exhibits high computational efficiency. As no complex convolutions or fully connected operations are required, Sim-CBS is computationally efficient and suitable for embedding into various CNN models. It exhibits significant performance improvements: experimental results demonstrate that Sim-CBS can markedly enhance the performance of CNNs in tasks such as image classification and object detection.

#### 2.2.3. NMS Loss Function and Non-Maximal Suppression Algorithm

The loss function for object detection consists of three parts: the predicted class loss Lossclass, the confidence loss Lossconf, and the bounding box loss Lossbbox for each predicted bounding box. Therefore, when constructing the loss function, it is necessary to evaluate the aforementioned components; calculate the class error, confidence error, and bounding box error of the prediction results separately; and obtain the overall loss function through weighting. The loss function for the predicted bounding box is defined as

(1) $$Loss={Loss}_{class}+{Loss}_{conf}+{Loss}_{bbox}$$

The bounding box loss, including calculation metrics like the GIoU, CIoU, DIoU, and EIoU, evaluates how well the predicted bounding box matches the ground truth. These loss functions typically assess factors such as the distance between the center points, the overlap area, or the aspect ratio between the predicted and ground truth boxes. However, none of them consider the direction between the ground truth bounding box and the predicted bounding box. This deficiency can lead to slower convergence speeds and lower efficiency of the model. In view of this, the SIoU loss function improves upon the traditional loss functions by considering the vector angle between the ground truth bounding box and the predicted bounding box during regression and redefining the penalty indicators. By adding an angle constraint to the predicted bounding box, the model can better align the predicted box with the ground truth, ensuring that it is either vertically or horizontally oriented, thus reducing the wobbling effect. Thus, during the training process of the model, it accelerates the convergence of the model and ultimately helps the model to train to achieve better results. Soft-NMS smooths the scores of overlapping boxes, preventing the issue of potential mis-suppression that can occur with traditional hard-NMS methods. It helps the model to retain more appropriate bounding boxes, reduces false positives, and optimizes box selection, thereby accelerating the convergence of the loss function and improving both the training efficiency and accuracy.

**Figure 6:**
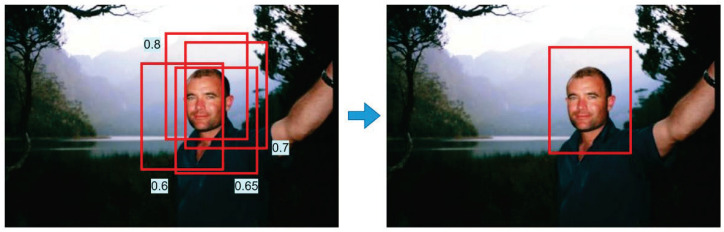
Ensuring the optimal prediction box.

**Figure 7:**
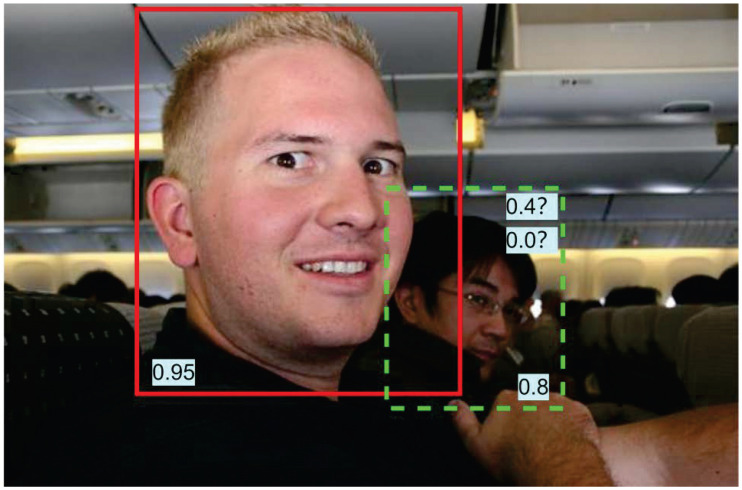
Comparison of predictive frame scores. The red box and the dashed green box represent different targets, and ? represents the possible score for the green box during detection.

Non-maximum suppression is an essential post-processing step in object detection algorithms, the purpose of which is to remove duplicate bounding boxes, i.e., to reduce false detection. In practical detection, the types of targets in complex scenes are diverse, and the background is also very cluttered. Moreover, the heads of pedestrians are relatively small, and, when there are many people, these heads may partially overlap. At this point, the complexity of the predicted boxes generated is even greater, so a good method is needed to eliminate this effect. At this point, if traditional NMS is used to process the bounding boxes, the NMS algorithm will set the scores of the bounding boxes that are greater than the threshold to zero, which can easily lead to the incorrect suppression of occluded objects and small objects, reducing the performance of the model. Therefore, we need to consider the overlapping area. The threshold may be set to 0.6, with the prediction frames exhibiting a probability greater than 0.6 retained. Subsequently, Soft-NMS is performed to retain the prediction frame that is most closely aligned with the bounding box, as illustrated in the accompanying Figure 6. The Soft-NMS approach considers both the degree of overlap and the score when performing NMS in order to achieve an optimal outcome. In the event of two objects within the same category exhibiting substantial overlap, even if both prediction frames possess high scores, only one can be retained under NMS. This is due to the considerable degree of overlap between the two prediction frames, as illustrated in Figure 7.

In contrast to the conventional approach of setting the original score to zero, the soft-NMS algorithm replaces it with a slightly lower score. Furthermore, soft-NMS can be readily integrated into the YOLO algorithm without necessitating the retraining of the original model. Accordingly, this paper employs the soft-NMS algorithm for non-maximal suppression.

The conventional non-maximal suppression algorithm initially generates a sequence of detection frames, designated as B, along with their respective scores, denoted as S. The detection frame M, exhibiting the highest score, is then removed from set B and incorporated into the final detection result set D. Concurrently, any detection frame within set B that exhibits an overlap with detection frame M exceeding the predefined overlap threshold Nt is also removed. The primary issue with the NMS algorithm is that it necessitates the assignment of a score of zero to all neighboring detection frames. In such instances, the presence of a genuine object within the overlapping region will result in the failure of its detection, thereby reducing the algorithm’s average precision (AP).

An alternative approach would be to reduce the scores of neighboring detection frames based on a correlation with the degree of overlap of M, rather than rejecting them completely. Despite the reduction in the score, the neighboring detection frames remain within the sequence of object detection.

## 3. Experiment Validation

### 3.1. Experimental Conditions

The experimental requirements include a substantial quantity of video data for the purpose of training and testing the head detection algorithm. Furthermore, high-performance computers are required to facilitate real-time image processing, object tracking algorithm execution, model evaluation, and optimization. It is therefore essential to establish an appropriate laboratory or experimental platform to facilitate research into object detection in complex scenes.

### 3.2. Experimental Dataset and Configuration

**Figure 8:**
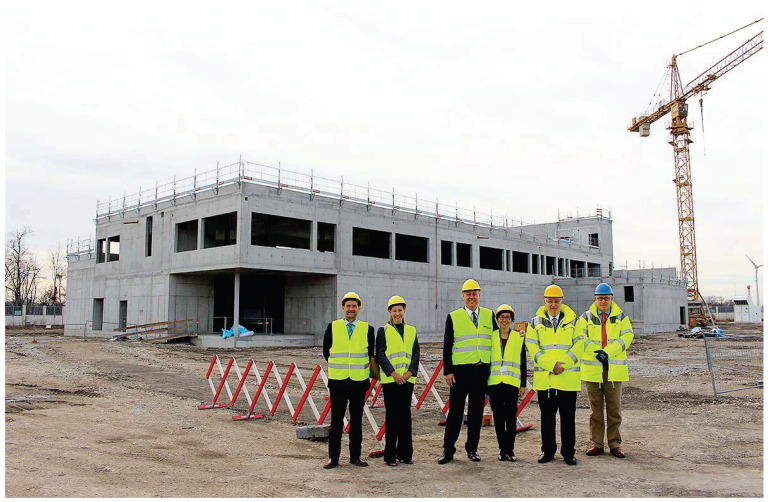
Example of VOC2012 dataset.

The dataset employed in this experiment is VOC2012 [35], a comprehensive object detection dataset. In this paper, we utilize the dataset’s category count for training purposes. The objective of this study is to examine pedestrian head detection in complex scenes. The VOC2012 dataset is distinguished by its uniformity of categories and the large number of instances compared to traditional datasets such as the COCO dataset. A fruitful avenue for research is head detection in complex scenes. The exclusion of a multitude of categories allows for a more focused and objected approach to research. This is the primary rationale for the selection of this dataset for the experiment, as illustrated in Figure 8.

The VOC2012 dataset, or the PASCAL Visual Object Classes Challenge 2012 dataset, is a widely used standard dataset in the field of computer vision. It is primarily employed for the evaluation and promotion of algorithmic performance in a range of tasks, including object recognition, classification, object detection, image segmentation, and other forms of visual understanding. The following section provides a comprehensive overview of the VOC2012 dataset.

#### 3.2.1. Dataset Content

The VOC2012 dataset comprises 20 categories of common everyday objects, including people (person), animals (e.g., cats, dogs, birds, etc.), vehicles (e.g., cars, bicycles, airplanes, etc.), and indoor objects (e.g., bottles, chairs, dining tables, etc.), in addition to backgrounds, resulting in a total of 21 categories. The dataset used in this work has been specifically curated for pedestrian head detection. It has been filtered to include only images with pedestrians, and the annotations focus solely on the heads of pedestrians. The dataset contains approximately 17,125 color images, which cover a wide range of complex scenarios, including roads, buildings, and indoor environments. Each image has been carefully annotated. These images feature challenging backgrounds and various factors that can complicate head detection, such as occlusion, varying lighting conditions, and cluttered environments. Pedestrians often overlap with other objects or blend into the background, making detection even more difficult.

Additionally, the diversity in pedestrians’ posture, angles, and actions further increases the detection difficulty. Pedestrians may appear in various poses or at different angles, which can lead to significant changes in their appearance and make them more difficult to recognize. To address this challenge, this work focuses on detecting the heads of pedestrians, which helps to avoid some of the difficulties caused by the occlusion of the full body. This localized detection approach reduces the impact of complex background and occlusion factors on the model, making the detection task more focused and accurate. Another challenge is the scale variation of pedestrians. In the images, the pedestrians may be at different distances from the camera, appearing smaller when farther away and larger when closer. Despite this, focusing on detecting pedestrian heads helps to mitigate the effects of scale changes, as the head is a more concentrated and recognizable feature compared to the whole body. This localized stability in scale allows the model to better adapt to variations in distance and size. By focusing on pedestrian head detection, this work effectively reduces the complexity arising from the posture, angle, and occlusion of the full body, ensuring that the model maintains high accuracy and stability when dealing with these diverse visual challenges.

#### 3.2.2. Dataset Preprocessing

We did not use the raw dataset and labels directly but performed some necessary processing. First, we filtered the dataset to create a new one containing images with human heads. Then, we processed the corresponding labels, keeping only those that indicated human heads. In the end, we obtained a dataset consisting solely of images of human heads and their labels. Training set: 70% of the data are used for the training process of the algorithm, including a large number of labeled images and their detailed information. Validation set: 20% of the data are used to validate the performance of the algorithm during training, allowing for adjustments. Test set: 10% of the data are used to evaluate the final performance of the algorithm, but the complete labels for the test set are not disclosed during the competition to ensure a fair performance evaluation.

### 3.3. Validation Set and Evaluation Criteria

The dataset used in this experiment is 10% of the VOC2012 dataset, as the validation set evaluation criteria for object detection are mainly used to measure the performance of object detection algorithms, which usually involves several aspects in order to comprehensively assess the algorithm’s performance in terms of accuracy, speed, and stability. The following are some of the evaluation criteria commonly used in object detection.

Precision: Precision specifically refers to the proportion of actual human heads among all instances predicted by the algorithm as “heads”. A high precision value indicates that the detection system can accurately identify more real targets, with fewer false detections. In complex scenes such as construction sites, head detection may face various challenges, such as changes in lighting, occlusion, and cluttered backgrounds. If an algorithm has high precision, it means that, in real-world applications, it can effectively reduce false alarms, thereby improving the efficiency and accuracy of subsequent processing (such as elevator monitoring, visitor management, etc.). This is significant for construction site safety, personnel flow management, and other aspects. The precision is calculated using the following formula:

(2) $$Precision=\frac{TP}{TP+FP}$$

where TP true positives, or the number of samples predicted to be positive that are actually positive; FP denotes false positives, or the number of samples predicted to be positive that are actually negative.

Recall: Recall, also known as sensitivity, is the proportion of actual positive instances that are predicted to be positive. In object detection, higher recall means that the algorithm detects more true positives and reduces the number of false negatives. Recall is calculated according to the following formula:

(3) $$Recall=\frac{TP}{TP+FN}$$

where FN denotes false negatives, or the number of samples predicted to be negative that are actually positive.

Mean Average Precision (mAP): On construction sites, there are typically different target categories (e.g., workers, visitors, security personnel, etc.), and we calculate the average of all categories’ AP to obtain the mAP. The mAP is an important evaluation metric in complex scenes for head detection, as it comprehensively assesses the effectiveness of detection algorithms in multi-category target recognition. The mAP@0.5 is the mean average precision at an IoU threshold of 0.50, and the mAP@0.95 is the mean average precision averaged over IoU thresholds from 0.50 to 0.95. Here, N is the total number of classes and $AP_{i}$ is the average accuracy for the i-th class.

(4) $$mAP=\frac{AP_{1}+AP_{2}+\cdots +AP_{N}}{N}$$

Giga floating point operations per second (GFLOPs) is a metric used to measure the computational complexity of a model, specifically in terms of how many floating point operations a model performs during inference. It provides insights into the amount of computational power required by a model to process inputs and produce outputs. In the context of object detection, GFLOPs is commonly used to evaluate the efficiency of the model’s computation. GFLOPs represents the number of floating point operations performed by the model per second. It is calculated as

(5) $$\mathrm{GFLOPs}=\frac{\mathrm{Total}\;\mathrm{Floating}\;\mathrm{Point}\;\mathrm{Operations}}{10^{9}}$$

where the total floating point operations term refers to the number of floating point operations required to process one inference.

In conclusion, the evaluation criteria for object detection encompass a range of factors, including the recall, AP, mAP, and so forth. In the elevator monitoring scenario within buildings, the performance evaluation of object detection algorithms typically requires the combination of multiple metrics to ensure that the system can accurately monitor and identify people and objects inside the elevator in real time. In elevator monitoring, the precision can help us to assess the system’s accuracy in identifying passengers, avoiding false positives. Recall is particularly important in elevator monitoring, especially when identifying a larger number of passengers, to ensure that the system does not miss any passenger. The mAP can also be calculated for different objects to ensure the system’s detection capabilities for various objects.

### 3.4. Ablation Experiments

The ablation experiments employed the category of heads in the pedestrian section of the VOC2012 dataset as the basis. In the context of object detection in complex scenes, the incorporation or alteration of specific components within the YOLOv8 framework can enhance the overall accuracy of the model.

This paper presents the design of a novel backbone network, Backbone-CENet. The network is principally based on two key modules, CESA and Sim-CBS, both introduced in this work. Each of these approaches offers distinctive advantages, enhancing the performance and effectiveness of the detection model through different technical means. CESA has the ability to enhance the contextual connections and perform scale correction. By strengthening the relationship between global features and local features, especially in complex scenes, CESA enhances the network’s ability to detect small objects. Scale correction, on the other hand, addresses the issue of inconsistent feature scales resulting from various convolutions. Without this step, subsequent convolutions may struggle to work effectively. Another module constructed in this paper, Sim-CBS, serves to enhance the convolution module. The Sim-CBS module was incorporated into the original backbone, and, in conjunction with the CESA module, a novel backbone network, designated Backbone-CENet, was devised. Following a series of experiments, it was demonstrated that the incorporation of Sim-CBS into the neck network yielded the most optimal results, although this approach did entail a certain degree of compromise in terms of the model’s overall performance. Additionally, this paper discusses the use of non-maximum suppression (NMS) to filter out duplicate frames. To guarantee the precision of the detection, the output frame of the detection network is typically denser. For each object, there will be multiple prediction frames. NMS is employed to filter out these duplicate frames, thereby retaining the optimal quality of the frame. Soft-NMS improves on traditional NMS by replacing the original score with a slightly reduced score, rather than zeroing it out. Furthermore, the soft-NMS approach can be readily incorporated into the YOLO algorithm, without necessitating the complete retraining of the original model. Accordingly, the soft-NMS algorithm is employed in this study for the purpose of suppressing non-extremely large values.

**Figure 9:**
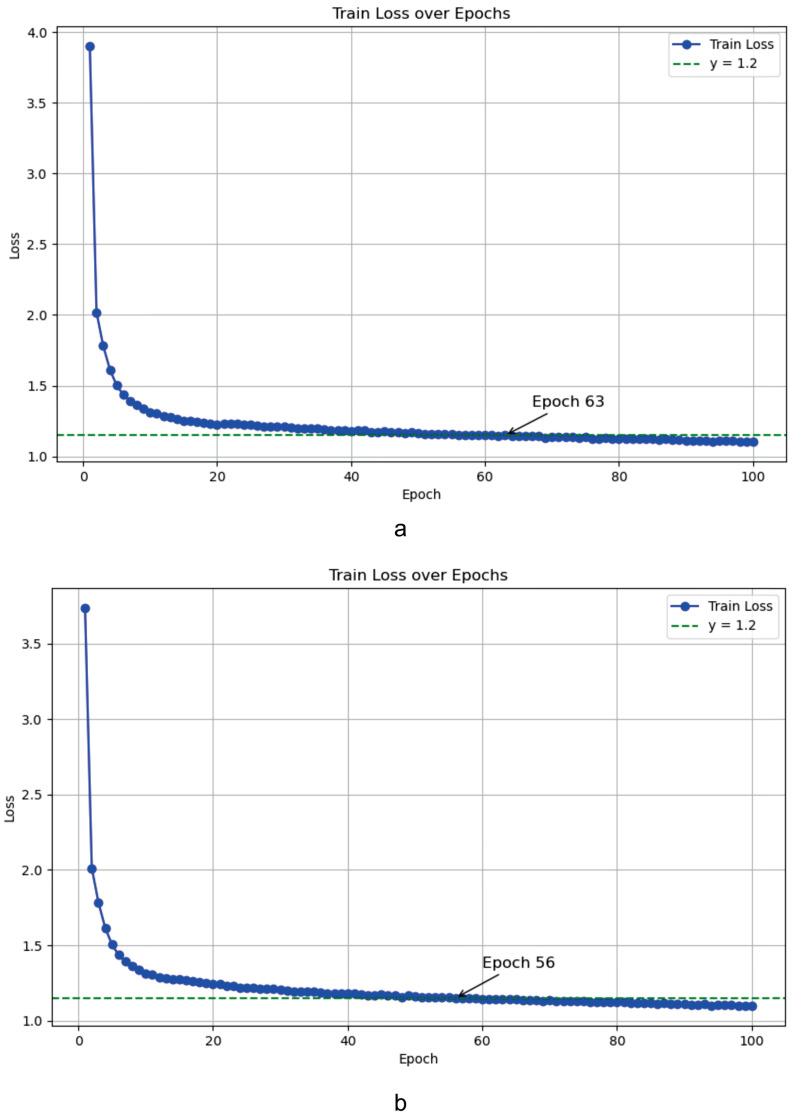
Train loss curve. (**a**) YOLOv8 train loss curve. (**b**) YOLO-HDCS train loss curve.

The incorporation of the CESA module as outlined in this paper will impact the model’s computation subsequent to its introduction. It can be foreseen that it will improve the efficiency of feature utilization, enhance the network’s perception ability, and accelerate the convergence speed of the model. Loss is a measure of the discrepancy between the model’s predictions and the true results during the prediction process. Specifically, the output of the loss function represents the degree of error in the model’s predictions. The smaller the loss, the better the model’s performance. Epoch refers to the process in which the model completes one forward and backward pass over the entire training dataset. One epoch represents a cycle of model parameter updates, and, typically, the training dataset is divided into multiple batches, with each batch containing a subset of the training samples. As shown in Figure 9, our loss converges faster, and, with the same loss value, fewer epochs are required. The enhancement of the multi-scale detection capabilities is contingent upon the fusion of disparate scale features within the network. In particular, some of the C2f blocks of the backbone in the original network architecture are replaced with CESA, while the other C2f blocks in the backbone are deliberately retained in order to maintain as much of the original information as possible during the initial processing. Furthermore, the average accuracy increased by 0.3% during the experimental phase. Integrating these two enhancements simultaneously into the detection network resulted in accuracy of 82.2%. The results of the ablation experiments are presented in Table 1.

The experimental results demonstrate that the improved detection network, YOLO-HDCS, exhibits enhanced accuracy and stability. The results also confirm the enhanced stability of the improved detection network model. This is sufficient to demonstrate that our improved method, YOLO-HDCS, has excellent detection capabilities and stability in complex environments. The significant improvement achieved on the VOC2012 dataset, which is complex and has a large number of objects, is in itself a great success.

### 3.5. Comparative Experiments

The same experimental configuration and VOC2012 dataset as in the ablation experiments were employed in the comparison experiments, and the experimental environment remained consistent. By demonstrating the comparison results with alternative models, the superiority of the proposed model can be more effectively proven, thereby enhancing the academic value and practical applicability of this paper. The object detection algorithm proposed in this paper demonstrates superior performance in complex scenes. The improved backbone network, Backbone-CENet, enables the model to have an extremely fast convergence speed and maintain a high level of stability in complex and variable scenes. This stability is crucial for practical applications, as it ensures the reliability and consistency of the algorithm in complex scenes. This is due to the improved module, CESA. To evaluate the performance of our head detection method, we compare our proposed YOLO-HDCS algorithm with the current mainstream object detection algorithms, such as SSD, Faster R-CNN, RT-DETR, and the YOLO series algorithms, under the same conditions. Table 2 shows that, in the tests, our method consistently outperformed the other methods by a significant margin. When compared with other methods, significant advantages are demonstrated. For instance, the proposed YOLO-HDCS model in this paper shows a GFLOPs value that is only 62.8% of that of the latest algorithm, RT-DETR [36], and it is superior when compared with the recently introduced YOLOX and YOLOv9 [37], two of the latest object detection algorithms. Therefore, even with a substantial reduction in GFLOPs, the accuracy remains superior. This fully demonstrates the excellence of the model’s performance. Although there is an increase in GFLOPs compared to YOLOX, the accuracy is improved by 17.5%. It is evident that the proposed YOLO-HDCS algorithm achieves the best balance between model performance and size, possessing strong practical value. The comparative experimental results also show that our method has powerful detection capabilities and stability in complex scenes, maintaining good performance even when there are a large number of targets and complex varieties.

## 4. Conclusions

In this paper, we propose a framework called YOLO-HDCS, based on a context-enhanced CNN, for head detection. The experimental results demonstrate that our approach consistently outperforms state-of-the-art methods in challenging head detection tests, achieving head detection in complex scenes, as well as small object detection in such scenes. The three main contributions to the performance improvement are the meticulously designed context enhancement and scale correction modules, the convolutional module based on an attention mechanism, and the soft-NMS algorithm. One of the main achievements is the design of a new backbone network, Backbone-CENet. This network is principally based on two key modules, CESA and Sim-CBS, both of which have been proposed in this paper. Each of these approaches offers distinctive advantages, enhancing the performance and effectiveness of the detection model through different technical means. The constructed module Sim-CBS is an improvement over the convolutional module. We inserted this Sim-CBS module into the original backbone and designed a new backbone network with the CESA module, called Backbon-CENet. Another improvement is enabled by NMS, which is a method of filtering out duplicate frames. To guarantee the precision of detection, the output frame of the detection network is typically denser. For each object, multiple prediction frames may be generated. NMS is employed to filter out these duplicate frames, thereby retaining the optimal quality of the frame. The soft-NMS algorithm replaces the original score with a slightly lower score, rather than setting it to zero. Furthermore, the soft-NMS approach can be readily integrated into the YOLO algorithm without necessitating the complete retraining of the original model. Accordingly, the soft-NMS algorithm was employed in this study for the purpose of non-maximal value suppression.

These enhancements led to optimal detection and reliability in the ablation and comparative experiments. Our proposed method demonstrates excellent performance and stability. Although detection in complex scenes is a challenging issue, our method can further address the problem of object detection in such complex environments and also provides insights and methods to solve the problems that arise when detecting small objects in complex environments. The work presented in this paper is distinctive and innovative, and it enhances the efficacy of object detection for head detection in complex scenes. In particular, this method significantly improves object detection in elevator scenes within buildings. Although our method is highly effective, further improvements can be made to enhance the network structure, particularly by incorporating more sophisticated attention mechanisms into each key model. Future work will focus on addressing additional challenges posed by increasingly complex scenes and refining our method for even better performance in dynamic environments.

As technology advances, a growing number of effective algorithms are being proposed, including the next iteration of the YOLO algorithm family. In terms of integration, it is more effective. Further enhancements can be made to the revised algorithms to optimize the network structure model for headcount detection in complex scenes. This may entail incorporating attention mechanisms into each of the key models. As the research progresses, more challenging improvements can be made within the module.

## Figures and Tables

**Table 1:** Ablation experiment results.

Method	mAP@0.5↑	mAP@0.95↑	Recall↑
YOLOv8s	0.805	0.505	0.705
YOLOv8s + Soft-NMS	0.818	0.560	0.718
YOLOv8s + CESA	0.808	0.506	0.706
YOLOv8s + Sim-CBS	0.811	0.549	0.711
YOLOv8s + Soft-NMS + CESA	0.820	0.561	0.722
YOLO-HDCS	0.822	0.565	0.724

**Table 2:** Comparative experiment results.

Method	mAP@0.5↑	mAP@0.95↑	GFLOPs↓
SSD	0.608	0.409	59
Faster R-CNN	0.623	0.409	23.8
YOLOv5m	0.640	0.406	49.0
YOLOX	0.647	0.409	26.8
YOLOv8s	0.805	0.505	28.8
RT-detr	0.777	0.485	60.0
YOLOv9s	0.804	0.585	26.4
Ours	0.822	0.565	37.7

## Data Availability

The data presented in this study are available on request from the corresponding author.

## Abbreviations

The following abbreviations are used in this manuscript:

YOLO	You Only Look Once
SSD	Single-Shot MultiBox Detector
YOLO-HDCS	YOLO-Based Head Detection in Complex Scenes
CNN	Convolutional Neural Network
CBS	Conv-BN-SiLU
Sim-CBS	Simple Attention Conv-BN-SiLU
CESA	Context Enhancement with Scale Adjustment Module
CAM	Context Augmentation Module
SimAM	SimpleAttention Module
IoU	Intersection over Union
VOC2012	PASCAL Visual Object Classes Challenge 2012
R-CNN	Region-Based Convolutional Neural Network
NMS	Non-Maximum Suppression
SIoU	Structured Intersection over Union
Faster R-CNN	Faster Region-Based Convolutional Neural Network
AP	Average Precision
TP	True Positive
FP	False Positive
FN	False Negative
mAP	Mean Average Precision
mAP@0.5	Mean Average Precision at IoU Threshold of 0.50
mAP@0.95	Mean Average Precision Averaged over IoU Thresholds from 0.50 to 0.95
